# Spatial Distribution of Inadequate Apparent Intake of Protein, Lysine, and Tryptophan among Malawi’s Households with Children Aged <5 y: Evidence from the 2019–2020 Integrated Household Survey

**DOI:** 10.1016/j.cdnut.2026.109448

**Published:** 2026-07-23

**Authors:** Keston Mwiwa Sinkhonde, Innocent Pangapanga-Phiri, Gareth Osman, Alexander A Kalimbira

**Affiliations:** 1 Département of Human Nutrition and Health, Bunda College, Lilongwe University of Agriculture and Natural Resources, Lilongwe, Malawi; 2 Centre for Agricultural Research and Development, Bunda College, Lilongwe University of Agriculture and Natural Resources, Lilongwe, Malawi

**Keywords:** amino acids, lysine, protein, spatial distribution, tryptophan

## Abstract

**Background:**

Understanding the spatial distribution of nutrient inadequacy is critical for designing context-specific interventions to improve human health.

**Objectives:**

The objective of our study was to determine the spatial distribution of inadequate apparent protein, lysine, and tryptophan intake among Malawi’s households with children aged <5 y.

**Methods:**

We retrieved household consumption and expenditure data for 5952 children aged 6 to 59 mo from Malawi’s 2019–2020 Integrated Household Survey under formal consent from the National Statistical Office. Relevant food composition tables and databases were used to estimate protein, lysine, and tryptophan intake based on food items. Using the adult female equivalent matrix, we applied Food and Agriculture Organization thresholds (0.66 g/kg/d protein, 35 mg/kg/d lysine, and 4.8 mg/kg/d tryptophan) to assess adequacy. Household consumption data were processed in R Studio. Median values were used to replace outliers and conversion factors utilized to compute edible portions. The processed data were merged with district shapefiles and subsequently analyzed to generate the spatial distribution of nutrient inadequacy (total and available protein, lysine, and tryptophan).

**Results:**

Regional disparities in nutrient inadequacy for total and available protein, lysine, and tryptophan were evident across Malawi, with inadequate available lysine intake being highly prevalent (>40%) in all districts in the Central Region, followed by the Southern Region, where 7 of 13 districts experienced lysine deprivation of >40%. Similarly, inadequate available protein intake was evident in the Central Region, followed by the Southern Region. The Northern Region exhibited the lowest inadequacy for total and available protein, lysine, and tryptophan intake among children.

**Conclusions:**

Regional variations in nutrient inadequacy were evident across Malawi, with the Central Region most affected by protein, lysine, and tryptophan inadequacy, followed by the Southern Region, although the Northern Region showed the lowest inadequacy. This highlights the need for region-specific interventions to address protein, lysine, and tryptophan inadequacy among children aged <5 y.

## Introduction

Childhood undernutrition is a serious public health concern with significant implications for children’s physical growth and cognitive development, ultimately affecting future work productivity and the overall national economy [1]. At the regional level, the prevalence of child stunting stands at 32.2% across Sub-Saharan Africa [1]. In Malawi, the prevalence of child stunting—an indicator of chronic inadequate intake of macronutrients and micronutrients compounded by recurrent infections and poor environment—is very high (38%), remaining at that level since 2015–2016 despite previous improvements [2]. Subnational geographical disparities in stunting are evident, being comparatively higher in the Southern (39%) and Central (38%) Regions than in the Northern Region (33%) [2]. Similarly, in Tanzania and Zambia, prevalence rates are 32% and 30%, respectively [3,4]. When assessed against the global standards, these figures are considered very high [5]. Inadequate dietary intake of essential nutrients is among the immediate causes of undernutrition, including child stunting [6].

Malawi, classified by the United Nations as one of the least developed countries, has an estimated population of 21.2 million, which is growing at 2.23% per annum, with a life expectancy of 67.9 y [7]. With a human development index of 0.517, which is classified as low, Malawi is ranked 172 of all countries in the world [8]. Rainfed agriculture is the key driver of the country’s economy, with tobacco being the main source of foreign exchange, accounting for ≤50% of exports [9]. Maize is Malawi’s dominant crop, occupying 50% of the country’s planted area [9] and providing 74% of dietary energy [10].

In Malawi, children’s diets remain inadequate. Only 24% of children aged 6 to 23 mo meet the minimum dietary diversity [2]. Dietary diversity remains low for most of the population, whose diets are dominated by cereal, which contributes ∼67% of total protein intake [10]. Cereals are poor sources of quality protein and minerals [11], and the nutritional quality is further compromised by antinutritional factors such as phytates, tannins, and trypsin inhibitors [12].

Proteins are compounds composed of hydrogen, carbon, nitrogen, oxygen, and sometimes sulfur atoms, arranged in chains of amino acids (AAs) [12]. They are distributed throughout the body, with >40% located in skeletal muscle, >25% in vital organs, and the remainder primarily in the skin and blood [13]. Proteins are essential for building and repairing tissues, regulating bodily processes as hormones and enzymes, and providing structural support and immune defense [12]. They are particularly critical for children in their rapid growth phase, where adequate intake of protein—especially essential AAs (EAAs) such as lysine and tryptophan—directly influences linear growth and overall development [14]. The quality of protein depends on the composition of EAAs [15], digestibility, and bioavailability [16]. Dietary intake of protein and EAAs lysine and tryptophan among infants and children is considered adequate when the mean dietary intake is 0.66 g/kg/d for protein, 35 mg/kg/d for lysine, and 4.8 mg/kg/d for tryptophan [17]. The values reflect the minimum daily needs for normal growth, body tissue function, and metabolic demand such that intakes less than these levels are classified as inadequate and may contribute to child stunting [18].

Lysine is an EAA that serves primarily as a building block for protein [19], necessitates energy production and carbohydrate metabolism [20], and facilitates the transport of long-chain fatty acids into mitochondria for energy generation [20]. Lysine also supports bone health by enhancing calcium absorption in the gut and reducing calcium loss through urine [20]. Beyond its role in protein synthesis, tryptophan contributes to the production of gastrointestinal serotonin essential for gut–brain axis neurotransmission that influences appetite. [21]. Lysine and tryptophan are the most deficient in cereals, particularly maize [22], which dominates Malawian diets [10,23]. Suboptimal supply of lysine and tryptophan not only affects protein synthesis but also disrupts metabolic pathways responsible for producing key biochemical compounds such as serotonin and melatonin, which are vital for appetite regulation and protection against oxidative stress [19,21].

Optimal dietary intake of quality protein, particularly animal source foods such as meat, eggs, and milk, provides a complete AA profile [14], is highly digestible and bioavailable [16], and has been associated with improved height-for-age in children [24]. A study in Rotterdam demonstrated that, unlike plant-based diets, animal-based protein sources were significantly associated with reduced odds of sarcopenia among adults (odds ratio: 0.62; 95% confidence interval: 0.43, 0.90; *P* = 0.01) [25]. Diets rich in animal protein are known to contain complete EAA profiles that are critical for the formation and maintenance of appendicular skeletal muscle [26]. A study in Malawi revealed that there was a high risk of inadequate dietary intake of protein and EAAs among the poorest households [27]. Although Sinkhonde et al. [18] found that apparent inadequate intake of protein, lysine, and tryptophan was highly prevalent among stunted children, those living in rural areas, and those residing in the Central Region, their study did not present the spatial distribution of these inadequacies by district and by region among Malawian children aged <5 y.

It is known that Malawi obtains 67% of its protein from cereals [10], with maize alone contributing 33.3% [10]. However, maize protein is deficient in lysine and tryptophan [28]. Inadequacy of these AAs has been associated with child stunting [29], which remains a major public health concern in Malawi [2]. Knowledge of the distribution at the regional and district levels is necessary, as dietary patterns and nutrient intake may vary substantially across Malawi due to geographic, environmental, and socioeconomic differences, thereby undermining contextual efforts that could curb the problem according to magnitude. Therefore, we aimed to determine the spatial distribution of inadequate apparent intake of total and available protein, lysine, and tryptophan among Malawian households with ≥1 children aged <5 y.

## Methods

We used data from Malawi’s Fifth Integrated Household Survey (IHS5), implemented from 2019 to 2020 across all 28 districts in Malawi, largely representing the rural stratum and 4 areas that represented the urban stratum. Supported by the World Bank and conducted every 3 to 7 y, the IHS5 is primarily used to monitor and evaluate progress in the fight against poverty and vulnerability among Malawian households and provides estimates on household consumption, expenditure, food security, child nutritional status, and agricultural activities [30].

The survey employed a cross-sectional design with a stratified 2-stage sampling approach, where enumeration areas (EAs; primary sampling units) were selected during the initial stage [30]. An EA is defined as the smallest census unit, delineated by fixed geographic boundaries, and typically comprises ∼235 households [30]. Due to coronavirus disease 2019 and nonresponse during the implementation period, 11,434 households were successfully interviewed of the 12,000 sampled across 750 EAs.

We retrieved the 2019–2020 IHS5 dataset from the World Bank Microdata Library (https://microdata.worldbank.org/index.php/catalog/3818) after formal consent from the Malawi National Statistical Office was granted on 18 December, 2024, in compliance with the data usage guidelines and conditions set forth by both institutions. The IHS5 collected household food consumption and expenditure data using a 7-d recall based on a list of 135 predefined food items. From the 11,434 surveyed households, a subsample of 5952 households with ≥1 child aged 6 to 59 mo was drawn.

### Statistical analysis

#### Preprocessing of household consumption data

We used region-specific conversion factors provided in the caloric conversion factor file in the IHS5 dataset to convert household consumption quantities apparently recorded in nonstandard units (e.g., basin, pail, plate) into standard units (e.g., kilograms), which were further reduced to grams by multiplying quantities in kilograms by 1000 g to derive consumption quantities in grams per household per week [31]. Further, consumption per week was divided by 7 to obtain consumption per day. We also applied the IHS5 edible portion conversion coefficients accessed from the World Bank Microdata Catalog (https://microdata.worldbank.org/index.php/catalog/3818) to compute edible portions of household food items inherently recorded to improve on representativeness of what was actually consumed at household level, in alignment with the study by Sinkhonde et al. [18]; for example, banana peels from ripe bananas and bones in meat are not edible [31]. We used log transformation to identify and manage outliers to estimate the normal distribution of data. Values exceeding +5 SDs were considered outliers and replaced with the median for corresponding food items, because the median is less affected by data skewness and provides a stable measure of central tendency [32,33]. In contrast, lower-end values were retained as they were more likely to represent actual observed levels.

#### Food matching

We employed the Food Composition Table (FCT) developed for the 2016–2017 Malawi Fourth Integrated Household Survey (IHS4) to assess nutrients from the household consumption and expenditure data, because the food item lists in IHS4 and IHS5 are broadly comparable [18,23]. Consistent with the study by Sinkhonde et al. [18], we compiled nutrient values on total and available protein. In summary, ∼72% of the nutrient values were obtained from the Malawian FCT 2019 [23]. Additionally, 26% of the nutrient values were obtained from the South African FCT to account for processed foods, such as biscuits, infant formulas, and beverages, that were not found in the Malawian FCT [34], although <3% of the nutrient values were drawn from the West African FCT [35] and the USDA FoodData Central Database [36]. Due to limited national and regional data on AAs, values for lysine and tryptophan were largely extracted from the USDA FoodData Central Database [18,23] with FCT items from Malawi and South Africa matched to comparable USDA entries to obtain lysine and tryptophan values as previously reported by Muleya et al. [23]. In this study, “total protein” refers to the complete protein content of a food item, while “available protein” reflects the fraction that is digestible and bioavailable for human metabolism [17]. Apparent available protein was calculated by matching each food item consumed to the digestibility coefficient of the respective food item consistent with the approach of Muleya et al. [23].

#### Calculation of adult female equivalent

For this study, apparent household food consumption was estimated and daily intake of protein, lysine, and tryptophan in grams per adult female equivalent (AFE) were calculated. The AFE approach assumes that the distribution of household food within the household is proportional to the energy requirement of each member, standardized to one reference individual: a nonpregnant, nonlactating female aged 18 to 29 y [33]. The same approach was used in a similar study by Sinkhonde et al. [18]. Energy requirements for each household member were estimated according to age, sex, physiological status (such as pregnancy or lactation), and average body weight [37]. For adults, moderate physical activity level factor of 1.6 applied to the basal metabolic rate was also taken into account [23]. We applied the mean body weight of 55 kg for females of reproductive age according to the 2015–2016 Malawi Demographic Health Survey [38]. For breastfed children, energy intake from complementary foods was estimated by subtracting the age-specific energy contribution of breastmilk from their total daily energy requirement [39]. This approach was also consistent with the study by Sinkhonde et al. [18]. An AFE for each household member was finally calculated by dividing their individual energy requirement by that of the reference adult female, and the total household AFE obtained by summing all individual values [18].

#### Analysis of apparent nutrient adequacy

We derived thresholds for protein, lysine, and tryptophan intake in children from the estimated average requirements (EARs) of a reference adult female (nonlactating, nonpregnant, aged 18 to 29 y): 36.3 g/d for protein, 1.95 g/d for lysine, and 0.26 g/d for tryptophan. Apparent nutrient intakes, standardized using the AFE approach, were compared against reference values to assess household-level risk of inadequacy. The reference values were based on FAO/WHO/UNU requirements: 0.66 g/kg/d for protein, 35 mg/kg/d for lysine, and 4.8 mg/kg/d for tryptophan [17,40]. To ensure comparability between household intakes expressed per AFE and the adequacy benchmarks, values were converted to AFE daily thresholds using the 55 kg reference body weight applied in the AFE framework. This yielded thresholds of 36.3 g/d for protein, 1.95 g/d for lysine, and 0.26 g/d for tryptophan [18,41]. Accordingly, thresholds were then used as household-level proxy indicators of apparent nutrient adequacy among households with ≥1 child aged <5 y. The term ‘apparent’ was used because the analysis of nutrient inadequacy relied on estimates rather than direct measurements of household consumption, consistent with the approach used by Kalimbira et al. [42]. The main outcome of the study was the spatial distribution of apparent nutrient inadequacy (total and available protein, lysine, and tryptophan) delineated at the subnational level by district (*n* = 28).

We computed prevalence estimates for total and available protein, lysine, and tryptophan with 95% confidence intervals to indicate statistical precision, allowing nutrient-based comparisons between apparent total and available intake. To address potential underreporting in Household Consumption and Expenditure Survey (HCES) data, we assessed both apparent energy intake (kilocalories per AFE) and nutrient density (per 1000 kilocalories) among eligible households for total protein, lysine, and tryptophan. Apparent energy intake was used to provide context for dietary adequacy. Nutrient density was defined as the ratio of each total nutrient supply to its total energy supply, expressed per 1000 kilocalories, allowing assessment of dietary quality independent of total energy intake [43]. Nutrient density was used as a sensitivity analysis to evaluate whether conclusions on protein and AA inadequacy were robust to variation in total energy reporting.

#### Spatial distribution analysis

We analyzed household consumption and expenditure data using Stata/SE version 17.0 for Windows (Revision 5 May, 2021; StataCorp LLC). The analysis focused on households with ≥1 child aged 6 to 59 mo and included data from 5952 households meeting this criterion, to estimate the prevalence of apparent nutrient inadequacy for total and available protein, lysine, and tryptophan. We then merged the nutrient inadequacy prevalence dataset with district-level Malawi shapefiles accessed from GeoPostcodes. We used ArcGIS (Esri) to generate spatial distribution maps of apparent nutrient inadequacy, illustrating district- and regional-level patterns for total and available protein, lysine, and tryptophan. The spatial maps were presented in pairs of total compared with available inadequate intake of protein, lysine, and tryptophan.

#### Ethical approval

The study protocols were reviewed and approved by the Lilongwe University of Agriculture and Natural Resources Research Ethics Committee (LUANAR-REC) on 4 April, 2025, under protocol number 2025-0012. In addition, informed consent for the use of IHS5 data was granted by the National Statistical Office, in adherence with all applicable data privacy and confidentiality regulations.

## Results

### Median apparent intake of total and available protein, lysine, and tryptophan with corresponding EARs

Table 1 shows the median (IQR) apparent intake of total and available protein, lysine, and tryptophan with corresponding EARs among households with children aged <5 y, stratified by region (*n* = 5952). The median intake of total protein was highest in the Northern Region, followed by the Southern Region, with the lowest intake observed in the Central Region. A similar regional pattern was observed for apparent total and available lysine and tryptophan (Table 1). When bioavailability was considered, apparent median intakes were consistently higher for total compared with available protein, lysine, and tryptophan. For both apparent total and available protein, lysine, and tryptophan, the median intakes generally exceeded the EAR, indicating that most households were meeting recommended intake levels. However, exceptions were noted for apparent available lysine, where the median intake in the Central Region and the overall national median were lower than the EAR, suggesting potential inadequacy. Furthermore, the wide IQRs highlight substantial disparities in household consumption, indicating that some households with children aged <5 y may remain at risk of inadequate intake despite overall adequacy at the population level.

**TABLE 1:** Median (IQR) apparent intake of total and available protein, lysine, and tryptophan with corresponding EARs among households with children aged <5 y, by region (*n* = 5952)

Geographic classification	Nutrient type	Intake category	Apparent Median intake (g/d/AFE)	IQR	EAR (g/d)
National	Protein	Total	54.76	38.34–78.50	36.3
		Available	44.81	31.34–64.58	36.3
	Lysine	Total	2.24	1.49–3.84	1.95
		Available	1.83	1.19–2.83	1.95
	Tryptophan	Total	0.49	0.33–0.73	0.26
		Available	0.40	0.27–0.60	0.26
Northern	Protein	Total	59.56	41.37–83.35	36.3
Central			49.30	35.06–73.27	36.3
Southern			56.84	40.51–80.32	36.3
Northern		Available	48.70	33.68–68.70	36.3
Central			40.62	28.48–60.34	36.3
Southern			46.76	33.12–66.10	36.3
Northern	Lysine	Total	2.61	1.74–3.86	1.95
Central			1.97	1.29–3.11	1.95
Southern			2.32	1.58–3.40	1.95
Northern		Available	2.12	1.43–3.25	1.95
Central			1.59	1.02–2.58	1.95
Southern			1.90	1.29–2.84	1.95
Northern	Tryptophan	Total	0.56	0.37–0.82	0.26
Central			0.44	0.30–0.68	0.26
Southern			0.51	0.35–0.73	0.26
Northern		Available	0.46	0.30–0.67	0.26
Central			0.36	0.24–0.56	0.26
Southern			0.41	0.28–0.59	0.26

The EARs were used to calculate prevalence of apparent inadequate intake of total and available protein, lysine, and tryptophan found in Table 2.

Abbreviations: AFE, adult female equivalent; EAR, estimated average requirement.

### Overall national prevalence of apparent protein, lysine, and tryptophan inadequacy

Table 2 shows an overall prevalence of apparent inadequate intake of total and available protein across the 3 regions of Malawi. The analysis revealed a higher prevalence of apparent inadequate dietary intake of available protein, lysine, and tryptophan compared with total protein and the 2 EAAs lysine and tryptophan. By region, the highest prevalence of inadequate intake of total and available protein as well as lysine and tryptophan were observed in the Central Region. This was followed by the Southern Region, while the Northern Region exhibited the lowest prevalence. In terms of specific nutrients, lysine had the highest rate of inadequacy, followed by protein, with tryptophan showing the lowest level of inadequacy. For detailed information on the prevalence of apparent inadequate intake of total and available protein, lysine, and tryptophan by district among children aged 6 to 59 mo in Malawi, along with the corresponding confidence intervals, see Supplemental Table 1.

**TABLE 2:** Prevalence of inadequate of total and available protein, lysine, and tryptophan among Malawian households with children aged <5 y (*n* = 5952)

Geographic classification		Protein		Lysine		Tryptophan	
		Total	Available	Total	Available	Total	Available
National		33.3 (30.0, 34.3)	48.2 (44.6, 50.8)	42.0 (37.3, 43.6)	54.4 (50.8, 5.1)	13.2 (11.0, 14.2)	24.1 (21.1 25.1)
Regional	Northern	30.8 (25.1, 30.6)	45.6 (38.8,4 9.7)	37.2 (28.5, 39.7)	47.8 (43.2, 49.2)	7.8 (5.8, 9.0)	19.1 (14.6, 19.1)
	Central	39.9 (37.3, 41.3)	53.2 (51.5, 55.7)	49.4 (47.3, 51.5)	61.5 (58.8, 62.9)	19.7 (17.4, 20.7)	31.5 (28.9, 32.7)
	Southern	29.7 (27.5, 30.9)	45.8 (42.4, 46.9)	39.5 (36.0, 39.6)	53.8 (50.4, 56.2)	11.3 (9.9, 12.2)	21.8 (19.8, 22.9)

Values represent the prevalence (%) of households with at least one child aged 6-59 months whose apparent intake of total or available protein, lysine, or tryptophan fell below the corresponding Estimated Average Requirement (EAR) threshold. Values in parentheses are 95% confidence intervals (CI). Apparent inadequacy was defined using thresholds of 36.3 g/day for protein, 1.95 g/day for lysine, and 0.26 g/day for tryptophan expressed per adult female equivalent (AFE).

### Spatial variation in apparent energy intake and nutrient density for protein, lysine, and tryptophan

Mean apparent energy intake per day per AFE varied across districts, ranging from ∼1758 to 2655 kcal/d per AFE, with most districts clustering between 1900 and 2300 kcal. Districts such as Dowa and Lilongwe (noncity) exhibited relatively lower energy availability, while districts such as Nkhata Bay and Zomba (city) showed higher estimated energy intake (Table 3). In contrast, nutrient density showed a different pattern of variation. Protein density ranged from 23.1 to 33.1 g/1000 kcal, while lysine density varied from ∼1.0 to 1.5 g/1000 kcal. Tryptophan density showed relatively little variation across districts (∼0.2–0.3 g/1000 kcal) (Table 3).

**TABLE 3:** Mean nutrient density (per 1000 kcal) and apparent energy intake per adult female equivalent (AFE) by district (95% confidence interval)

District	Protein (g/1000 kcal)	Lysine (g/1000 kcal)	Tryptophan (g/1000 kcal)	Energy (kcal/d/AFE)
Chitipa	31.0 (30.1, 31.9)	1.3 (1.3, 1.4)	0.3 (0.3, 0.3)	2296.0 (2178.3, 2413.7)
Karonga	28.1 (26.7, 29.5)	1.3 (1.2, 1.4)	0.3 (0.3, 0.3)	2159.3 (2019.2, 2299.3)
Likoma	28.5 (25.1, 31.9)	1.4 (1.1, 1.7)	0.3 (0.2, 0.3)	2655.4 (1945.2, 3365.5)
Mzimba	30.4 (29.6, 31.2)	1.4 (1.3, 1.4)	0.3 (0.3, 0.3)	1911.0 (1794.7, 2027.2)
Mzuzu City	30.2 (29.2, 31.2)	1.5 (1.4, 1.6)	0.3 (0.3, 0.3)	2390.3 (2097.0, 2683.5)
Nkhata Bay	23.1 (20.7, 25.5)	1.1 (1.0, 1.3)	0.2 (0.2, 0.3)	2528.7 (2256.5, 2800.8)
Rumphi	30.4 (28.4, 32.3)	1.3 (1.3, 1.4)	0.3 (0.3, 0.3)	2449.7 (2307.3, 2592.1)
Dedza	31.6 (30.9, 32.3)	1.3 (1.2, 1.4)	0.3 (0.3, 0.3)	1853.7 (1688.4, 2019.0)
Dowa	31.7 (30.5, 32.9)	1.3 (1.2, 1.4)	0.3 (0.3, 0.3)	1758.5 (1572.2, 1944.8)
Kasungu	33.1 (31.6, 34.5)	1.4 (1.3, 1.6)	0.3 (0.3, 0.3)	1909.0 (1765.8, 2052.2)
Lilongwe noncity	31.6 (30.9, 32.4)	1.3 (1.2, 1.4)	0.3 (0.3, 0.3)	1848.7 (1738.4, 1959.0)
Lilongwe city	31.1 (30.5, 31.7)	1.5 (1.4, 1.5)	0.3 (0.3, 0.3)	2087.8 (1970.7, 2204.9)
Mchinji	33.1 (31.2, 35.0)	1.4 (1.2, 1.6)	0.3 (0.3, 0.3)	1846.5 (1697.9, 1995.2)
Nkhotakota	24.6 (22.6, 26.7)	1.0 (0.9, 1.1)	0.2 (0.2, 0.2)	2128.5 (1986.4, 2270.6)
Ntcheu	31.1 (30.3, 31.9)	1.3 (1.2, 1.4)	0.3 (0.3, 0.3)	2071.1 (1915.4, 2226.9)
Ntchisi	32.0 (30.8, 33.2)	1.3 (1.2, 1.5)	0.3 (0.3, 0.3)	1962.3 (1838.4, 2086.1)
Salima	31.0 (29.8, 32.2)	1.3 (1.2, 1.4)	0.3 (0.3, 0.3)	1933.7 (1646.9, 2220.6)
Balaka	30.9 (30.0, 31.9)	1.3 (1.2, 1.4)	0.3 (0.3, 0.3)	1843.5 (1686.2, 2000.9)
Blantyre noncity	30.8 (30.1, 31.5)	1.3 (1.3, 1.4)	0.3 (0.3, 0.3)	2226.3 (2034.0, 2418.7)
Blantyre city	30.7 (29.3, 32.0)	1.5 (1.3, 1.6)	0.3 (0.3, 0.3)	2215.3 (2054.1, 2376.6)
Chikwawa	31.2 (30.6, 31.8)	1.2 (1.1, 1.3)	0.3 (0.3, 0.3)	1961.4 (1746.9, 2175.8)
Chiradzulu	31.3 (30.4, 32.2)	1.3 (1.3, 1.4)	0.3 (0.3, 0.3)	2310.3 (2106.2, 2514.4)
Machinga	30.3 (29.5, 31.0)	1.3 (1.2, 1.3)	0.3 (0.3, 0.3)	1867.8 (1745.1, 1990.4)
Mangochi	29.5 (28.2, 30.8)	1.2 (1.1, 1.3)	0.3 (0.3, 0.3)	1961.6 (1808.5, 2114.8)
Mulanje	29.0 (27.9, 30.0)	1.2 (1.1, 1.3)	0.3 (0.2, 0.3)	2172.7 (1973.5, 2371.9)
Mwanza	30.5 (29.7, 31.2)	1.3 (1.2, 1.4)	0.3 (0.3, 0.3)	1784.5 (1601.8, 1967.1)
Neno	31.2 (30.5, 31.9)	1.4 (1.3, 1.4)	0.3 (0.3, 0.3)	2032.1 (1902.1, 2162.0)
Nsanje	31.6 (30.6, 32.6)	1.3 (1.2, 1.4)	0.3 (0.3, 0.3)	2148.5 (1948.2, 2348.7)
Phalombe	31.5 (30.7, 32.3)	1.4 (1.3, 1.5)	0.3 (0.3, 0.3)	2151.4 (2034.1, 2268.8)
Thyolo	29.3 (28.5, 30.1)	1.2 (1.2, 1.3)	0.3 (0.2, 0.3)	2293.5 (2176.9, 2410.1)
Zomba city	30.8 (29.8, 31.8)	1.5 (1.4, 1.6)	0.3 (0.3, 0.3)	2559.4 (2374.9, 2743.9)
Zomba noncity	30.9 (29.7, 32.1)	1.3 (1.2, 1.4)	0.3 (0.3, 0.3)	2288.7 (2070.6, 2506.8)

Importantly, higher energy intake did not consistently correspond with higher nutrient density. For example, Nkhata Bay exhibited relatively high energy intake alongside comparatively lower protein density. Because nutrient density is expressed relative to total energy intake, this indicates that the composition of the diet rather than total energy alone differs across districts. Similarly, districts with comparable levels of energy intake displayed differences in nutrient density. These findings indicate that spatial differences in dietary adequacy reflect not only variation in overall energy availability but also differences in diet composition.

### Spatial distribution of inadequate apparent intake of total and available protein

**FIGURE 1:**
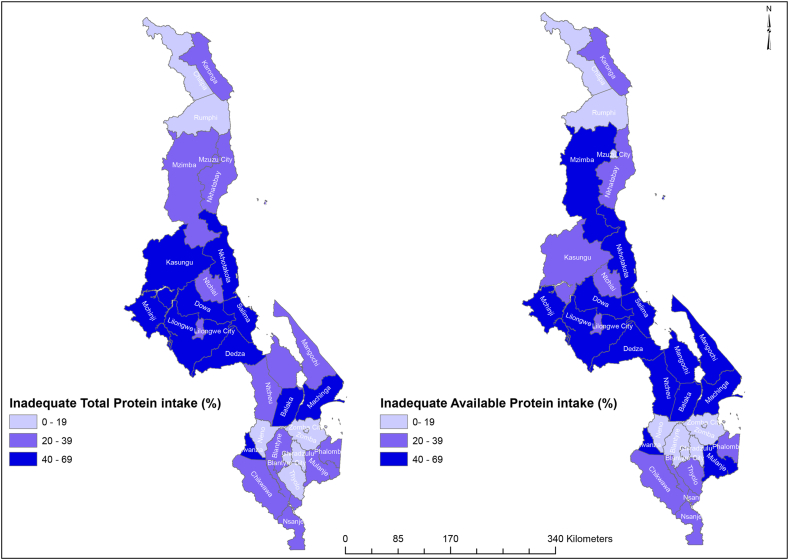
Spatial distribution of inadequate apparent intake of total and available protein.

Prevalence of inadequate apparent intake of protein was categorized into shared ranges of 0 to 19%, 20% to 39%, and 40% to 69% for both total and available protein. We observed notable disparities in the prevalence of apparent inadequate intake of available compared with total protein among households with ≥1 child aged <5 y (Figure 1). For example, in Mzimba, Mangochi, and Mulanje, the prevalence of protein inadequacy was higher for available protein (40%–69%) than for total protein (20%–39%). Across regions, the Central Region showed >40% inadequacy for both apparent total and available protein intakes. In the Southern Region, over 30% of households with ≥1 child aged <5 y experienced apparent available protein inadequacy compared with 20% total protein inadequacy. In the Northern Region, Karonga, Mzimba, and Nkhata Bay districts had the highest prevalence, with 20% to 39% for both total and available protein inadequacy.

### Spatial distribution of inadequate total and available lysine

**FIGURE 2:**
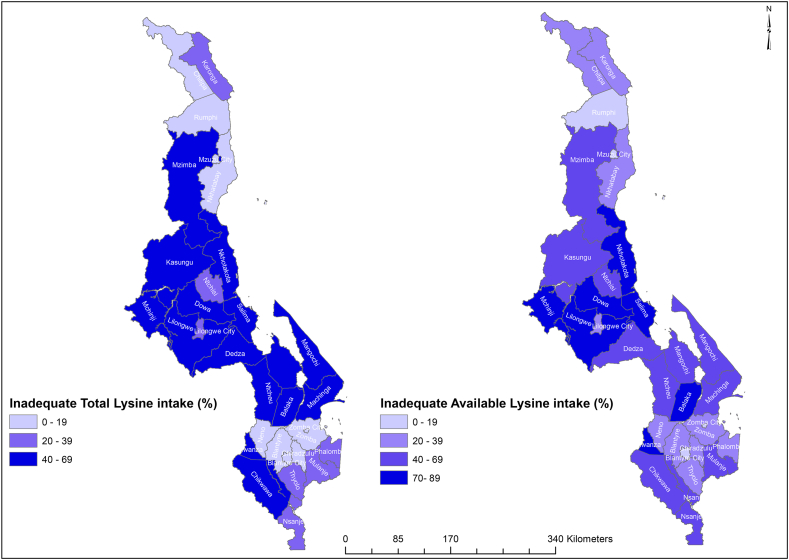
Spatial distribution of inadequate apparent intake of total and available lysine.

Figure 2 presents the distribution of inadequate apparent total and available lysine intake among Malawian households with children aged <5 (*n* = 5952). Overall, the prevalence of inadequate available lysine was significantly higher (20%–89%) than inadequate total lysine (20%–69%). Regionally, all districts (*n* = 9) in the Central Region reported available lysine inadequacy >40% to 89%, alongside total lysine inadequacy >40% to 69%. In the Southern Region, 7 of the 13 districts—Balaka, Machinga, Mangochi, Mwanza, Chikwawa, Nsanje, and Mulanje—recorded a high prevalence of inadequate available lysine (>40%), while 5 districts also reported apparent total lysine inadequacy >40%. In the Northern Region, Rumphi recorded the lowest prevalence of apparent inadequate intake of available lysine (0%–19%), while for total lysine, 3 districts—Nkhata Bay, Rumphi, and Chitipa—fell within the lowest inadequacy range of 0% to 19%.

### Spatial distribution of apparent inadequate total and available tryptophan

**FIGURE 3:**
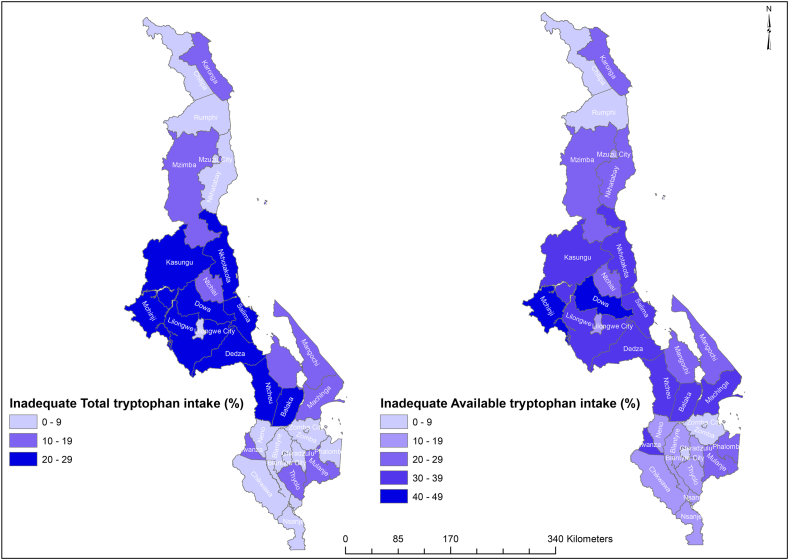
Spatial distribution of inadequate apparent intake of total and available tryptophan.

Figure 3 presents the prevalence of apparent inadequate total and available tryptophan intake among households with ≥1 child younger than 5 y across Malawi (*n* = 5952). The findings revealed a higher prevalence of inadequate available tryptophan intake, reaching ≤49%, compared with apparent inadequate total tryptophan intake of ≤29% across most districts in Malawi. Consistent with earlier results on protein and lysine, all districts (*n* = 9) in the Central Region reported available tryptophan inadequacy ranging from 20% to 49%, while total tryptophan inadequacy fell within the 10% to 29% range. Similarly, in the Southern Region, 5 of 13 districts reported a prevalence of inadequate available tryptophan intake ranging from 20% to 39%, while total tryptophan inadequacy was within the 10% to 29% range. In the Northern Region, 3 of 5 districts reported available tryptophan inadequacy in the 0% to 19% range, while 2 of 5 districts showed total tryptophan inadequacy within similar levels (10%–29%).

## Discussion

### Spatial distribution of apparent inadequate intake of total and available protein, lysine, and tryptophan among Malawian children aged <5 y

We found a high prevalence of apparent inadequate intake of total and available protein, lysine, and tryptophan among households with ≥1 child aged 6 to 59 mo (FIGURE 1, FIGURE 2, FIGURE 3). By focusing on these households, the analysis directly targeted the population most at risk of malnutrition, including stunting, given the high burden of undernutrition among young children in Malawi [44]. These findings may be linked to the predominance of plant-based protein sources in Malawian diets [23]. In Tanzania, malnutrition is also a major concern, with 32% of children experiencing stunting [4], largely linked to inadequate intake of macronutrients—especially quality protein among children and females of reproductive age due to diets that are dominated by maize and rice [45]. Evidence reveals that plant-based sources of protein are associated with an increased prevalence of protein inadequacy among households in low- and middle-income countries [46] because of antinutritional factors (e.g., trypsin inhibitor) found in plant foods—especially legumes—which interfere with protein digestion and absorption [46]. It is known that trypsin inhibitors, phytates, and tannins are common antinutritional factors found in cereals and legumes that interfere with protein and mineral digestibility and absorption [22]. A study in Malawi found a limited supply of dietary protein and the EAAs lysine and tryptophan among Malawian households, particularly in rural areas [23]. The study indicated that cereals, including maize supplied >66% of the total protein in Malawi [23], which are known to have incomplete protein [22]. Additionally, apparent inadequate intake of total and available protein, lysine, and tryptophan is highly prevalent in Malawi, with stunted children, those residing in rural areas, and those in the Central Region experiencing the highest burden [18].

Blending cereals with pulses is known to improve overall protein quality in animal models compared with cereals alone. This is because cereals such as maize are deficient in lysine, whereas legumes are rich in this EAA [47]. Furthermore, an estimated intake of 100 g/d of quality protein maize has been calculated to meet 100% of the WHO requirements for protein, lysine, and tryptophan among children [48]. This underscores the need for protein complementation, consumption of quality protein maize and animal foods to address protein inadequacy among children in Malawi. Additionally, enhancing home-based food processing techniques—such as soaking, germination, and fermentation—can significantly improve digestibility and absorption of protein and AAs from the widely consumed maize [49].

Geographically, we found a higher prevalence of inadequate intake of total and available protein, lysine, and tryptophan among households with children younger than 5 y in the Central Region compared to their counterparts in the Southern and Northern Regions. Regional economic disparities may contribute to variations in nutrient inadequacy among children under age 5 in Malawi. For example, poverty is most prevalent in the Central Region (55.8%), followed by the Southern Region (51.0%), while the Northern Region has the lowest poverty rate at 32.9% [50]. Similarly, regional disparities in the attainment of minimum acceptable diet (MAD), where the Central and Southern Regions have the lowest rates of attainment of MAD (8.6% and 8.3%, respectively), while Northern Region exhibited the highest (10.0%) [38]. Child food poverty is also not evenly distributed across Malawi. For example, 84.7% of children in the Central Region experienced child food poverty, followed by 83.7% in the Southern Region, while the Northern Region had the lowest prevalence at 70.6% [51]. This suggests that economically deprived households in the Central Region are less likely to afford high quality protein from animal sources; instead, plant-based sources of protein are an affordable option [23]. Mainstreaming nutrition education into existing social safety net programs (e.g., mtukula pakhomo, public works programs) would help in improving economic access to quality protein-rich foods among children younger than 5 y in Malawi.

A study conducted among Korean adults aged ≥19 y found a significant link between low protein intake and reduced muscle mass. This decline in muscle mass became more pronounced with decreasing socioeconomic status, with individuals in the lowest wealth quartile (quartile 1) experiencing the greatest reduction compared with those in the highest quartile (quartile 4) (*P* < 0.001) [52]. Lysine deficiency impairs growth and body compartment development, decreases albumin production, and elevates C-reactive protein (CRP) expression in the liver, independently of the proinflammatory cytokines—the main precursors of CRP [53]. The findings suggest that inadequate lysine intake is one of the driving factors of noncommunicable diseases among low socioeconomic communities, including Malawi, where diets are predominantly cereal-based [23,53].

Protein inadequacy alongside sedentary lifestyle is associated with sarcopenic obesity (SO) —a coexistence of obesity and low muscle mass and strength affecting the quality of life among adults [54]. Although SO is most evident in adulthood, it is rooted in childhood malnutrition that contributes to long-term consequences that manifest later in life [1]. However, this pattern poses a challenge to human capital development—a critical enabler in the context of decentralization and the principle of leaving no one behind [55]. Addressing this issue is essential for Malawi to achieve its vision of becoming an inclusive, wealthy, and self-reliant industrialized upper-middle-income country by 2063, while also advancing the 2030 Sustainable Development Agenda [55]. This underscores the need for integrating nutrition education into existing priority programs such as Village Savings and Loan schemes and Climate-Smart Public Works programs to strengthen economic access to nutrient-dense foods and address protein and AA inadequacy as described in the 2025–2030 National Multisector Nutrition Strategic Plan [45].

### Strengths and limitations of the study

To our knowledge, this study is the first to map the spatial distribution of protein, lysine, and tryptophan inadequacy across all districts in Malawi. The use of a nationally representative dataset strengthens the generalizability of the findings at the national level. Moreover, assessing nutrient inadequacy primarily through the Malawi FCT, supplemented with a limited number of external FCT entries, adds credibility to the findings. Nonetheless, dietary intakes were estimated using a 7-d dietary recall method, which introduces potential recall bias. In addition, the use of the AFE method to estimate apparent dietary intake represents another limitation, as the findings are based on approximations rather than direct measurements. Furthermore, the assessment of lysine and tryptophan using values derived from the USDA database constitutes an additional constraint, although those values are the best available. Moreover, we did not adjust for moisture content because most household surveys, including Malawi’s HCES, do not provide sufficient detail to make such adjustments; therefore, we followed the standard approach of using foods as reported in the household acquisition module, as previously described by Tang et al. [33]. We also acknowledge that the IHS5 data does not provide information on food preparation or cooking method.

To address potential underreporting in HCES data, we examined both apparent energy intake per AFE and nutrient density (per 1000 kcal). Energy estimates fell within plausible ranges for most districts and showed moderate variation. Importantly, nutrient density analysis produced broadly consistent spatial patterns, with districts exhibiting differences in dietary composition that were not explained by variation in total energy intake alone. These findings suggest that spatial variation in AA adequacy reflects both dietary quantity and composition, supporting the robustness of the results to potential reporting bias.

### Conclusion

This study highlights regional disparities of nutrient inadequacy among households with ≥1 child aged <5 y in Malawi, with the Central Region exhibiting the highest prevalence of protein, lysine, and tryptophan inadequacy, followed by the Southern Region, whereas the Northern Region had the lowest. The findings call for context-specific policy actions such as promoting protein complementation and consumption of quality protein maize and animal foods. Additionally, enhancing home-based food processing techniques such as soaking, germination, and fermentation can significantly improve digestibility and absorption of protein and AAs from the widely consumed maize. Integrating nutrition into safety net programs for vulnerable households to improve economic access to high quality protein-rich foods, such as those from animal sources, and mainstreaming nutrition education across food systems are necessary to drive sustained behavior change.

## Author contributions

The authors’ responsibilities were as follows – KMS, AAK: conceptualized the study; KMS: retrieved and managed the data, conducted the analysis, interpreted the findings, and drafted the manuscript; GO: assisted with data analysis; AAK, IP-P, GO: reviewed and edited both the draft and final versions of the manuscript; and all authors: read and approved the final manuscript.

## Data availability

The household consumption and expenditure data, forming part of the Fifth Integrated Household Survey (IHS5), can be accessed from World Bank’s microdata repository at https://microdata.worldbank.org/index.php/catalog/3818**.** For further details on the original contributions of this study, please contact the corresponding author.

## Declaration of Generative AI and AI-assisted technologies in the writing process

The authors declare that no generative AI or AI-assisted technologies were used in the writing of this manuscript.

## Funding

The authors reported no funding received for this study.

## Conflict of interest

The authors report no conflicts of interest.
