# Supplementary material for: Spatial Distribution of Inadequate Apparent Intake of Protein, Lysine, and Tryptophan among Malawi’s Households with Children Aged <5 y: Evidence from the 2019–2020 Integrated Household Survey
Source: Curr Dev Nutr. 2026 Jul 23;10(8):109448. doi: 10.1016/j.cdnut.2026.109448 (PMC13487606; doi:10.1016/j.cdnut.2026.109448)
Supplement: Supplementary file 1 [file mmc1.docx]

Supplementary table 1 Prevalence of inadequate apparent intake of total and available protein, lysine and tryptophan by district (95% CI)

| District | | Protein | | | | Lysine | | | | Tryptophan | | | |
| --- | --- | --- | --- | --- | --- | --- | --- | --- | --- | --- | --- | --- | --- |
|  |  | Total | | available | | Total | | available | | Total | | available | |
| Chitipa | | 17.5 (16.5–18.4) | | 31.1 (29.9–32.2) | | 25.9 (24.8–27.1) | | 41.2 (39.9–42.4) | | 4.4 (3.9–5.0) | | 10.7 (9.9–11.5) | |
| Karonga | | 34.9 (33.7–36.1) | | 47.4 (46.1–48.6) | | 34.9 (33.7–36.1) | | 45.4 (44.1–46.6) | | 9.8 (9.0–10.5) | | 20.4 (19.4–21.4) | |
| Likoma | | 29.1 (27.9–30.3) | | 53.2 (51.9–54.4) | | 29.1 (27.9–30.3) | | 34.1 (32.9–35.3) | | 10.1 (9.3–10.8) | | 10.1 (9.3–10.8) | |
| Mzimba | | 36.7 (35.5–37.9) | | 55.3 (54.1–56.6) | | 50.4 (49.1–51.7) | | 56.4 (55.1–57.7) | | 10.7 (9.9–11.4) | | 24.6 (23.5–25.7) | |
| Mzuzu City | | 28.4 (27.2–29.5) | | 34.6 (33.4–35.8) | | 27.2 (26.0–28.3) | | 34.6 (33.4–35.8) | | 2.5 (2.1–2.9) | | 14.5 (13.6–15.4) | |
| Nkhata Bay | | 31.6 (30.4–32.8) | | 49.0 (47.7–50.3) | | 28.0 (26.9–29.2) | | 46.9 (45.6–48.1) | | 7.7 (7.0–8.3) | | 21.7 (20.6–22.7) | |
| Rumphi | | 14.5 (13.6–15.3) | | 20.6 (19.6–21.6) | | 17.9 (16.9–18.9) | | 35.0 (33.8–36.2) | | 1.0 (0.8–1.3) | | 3.4 (2.9–3.8) | |
| Dedza | | 39.4 (38.1–40.6) | | 52.3 (51.0–53.5) | | 51.1 (49.8–52.4) | | 63.0 (61.8–64.3) | | 20.2 (19.2–21.3) | | 35.6 (34.4–36.8) | |
| Dowa | | 54.1 (52.8–55.4) | | 64.9 (63.7–66.1) | | 62.5 (61.2–63.7) | | 72.6 (71.5–73.7) | | 26.9 (25.7–28) | | 43.2 (42–44.5) | |
| Kasungu | | 39.6 (38.3–40.8) | | 50.0 (48.8–51.3) | | 48.1 (46.8–49.3) | | 60.3 (59.1–61.6) | | 20.3 (19.3–21.4) | | 30.8 (29.6–31.9) | |
| Lilongwe | | 38.3 (37.1–39.5) | | 53.7 (52.4–54.9) | | 51.7 (50.5–53.0) | | 66.1 (64.9–67.3) | | 21.8 (20.7–22.8) | | 32.7 (31.5–33.9) | |
| Lilongwe City | | 27.6 (26.5–28.8) | | 41.9 (40.7–43.2) | | 33.0 (31.8–34.2) | | 42.4 (41.2–43.7) | | 7.6 (6.9–8.3) | | 15.5 (14.5–16.4) | |
| Mchinji | | 48.3 (47.0–49.6) | | 60.7 (59.5–62.0) | | 58.4 (57.1–59.6) | | 66.8 (65.6–68.0) | | 26.3 (25.2–27.4) | | 40.3 (39.0–41.5) | |
| Nkhotakota | | 44.1 (42.8–45.3) | | 59.9 (58.6–61.1) | | 51.5 (50.3–52.8) | | 66.3 (65.1–67.5) | | 19.2 (18.2–20.2) | | 30.2 (29.0–31.3) | |
| Ntcheu | | 36.3 (35.1–37.5) | | 52.8 (51.5–54.1) | | 45.5 (44.2–46.8) | | 59.6 (58.4–60.9) | | 19.3 (18.3–20.3) | | 30.1 (28.9–31.3) | |
| Ntchisi | | 28.6 (27.5–29.8) | | 43.2 (41.9–44.4) | | 43.1 (41.8–44.3) | | 53.5 (52.3–54.8) | | 14.4 (13.5–15.3) | | 25.9 (24.8–27.0) | |
| Salima | | 44.7 (43.5–46.0) | | 59.6 (58.4–60.9) | | 54.2 (52.9–55.4) | | 66.1 (64.9–67.3) | | 21.6 (20.5–22.6) | | 33.5 (32.3–34.7) | |
| Balaka | | 44.9 (43.6–46.1) | | 57.6 (56.3–58.8) | | 51.6 (50.4–52.9) | | 68.9 (67.7–70.1) | | 20.7 (19.7–21.8) | | 35.5 (34.3–36.7) | |
| Blantyre | | 25.1 (24.0–26.2) | | 35.1 (33.9–36.3) | | 29.7 (28.6–30.9) | | 43.8 (42.5–45.0) | | 9.4 (8.7–10.2) | | 18.2 (17.2–19.1) | |
| Blantyre City | | 20.0 (19.0–21.0) | | 29.6 (28.5–30.8) | | 23.2 (22.1–24.2) | | 34.1 (32.9–35.3) | | 6.0 (5.4–6.6) | | 11.3 (10.5–12.1) | |
| Chikwawa | | 28.7 (27.5–29.8) | | 46.4 (45.1–47.6) | | 46.2 (44.9–47.4) | | 61.6 (60.4–62.9) | | 7.8 (7.1–8.5) | | 14.6 (13.7–15.5) | |
| Chiradzulu | | 22.6 (21.5–23.7) | | 35.1 (33.9–36.3) | | 27.6 (26.5–28.7) | | 39.8 (38.6–41.1) | | 6.3 (5.7–6.9) | | 17.3 (16.4–18.3) | |
| Machinga | | 42.9 (41.6–44.1) | | 56.6 (55.4–57.9) | | 49.9 (48.6–51.2) | | 62.8 (61.6–64.0) | | 17.9 (16.9–18.9) | | 34.8 (33.6–36.0) | |
| Mangochi | | 35.7 (34.5–36.9) | | 55.5 (54.2–56.8) | | 48.7 (47.4–50.0) | | 63.1 (61.8–64.3) | | 12.8 (12.0–13.7) | | 26.2 (25.1–27.3) | |
| Mulanje | | 31.4 (30.2–32.6) | | 53.8 (52.6–55.1) | | 42.7 (41.5–44.0) | | 59.3 (58.0–60.5) | | 13.2 (12.3–14.1) | | 24.5 (23.4–25.6) | |
| Mwanza | | 40.3 (39.1–41.6) | | 59.6 (58.3–60.8) | | 48.0 (46.7–49.2) | | 65.2 (64.0–66.4) | | 14.4 (13.5–15.3) | | 32 (30.8–33.2) | |
| Neno | | 22.5 (21.4–23.6) | | 32.9 (31.7–34.1) | | 27.3 (26.1–28.4) | | 40.0 (38.8–41.3) | | 8.5 (7.8–9.3) | | 17.9 (16.9–18.8) | |
|  | Protein | | | | Lysine | | | | Tryptophan | | | |  |
|  | Total | | available | | Total | | available | | Total | | available | |  |
| Nsanje | 29.6 (28.4–30.8) | | 42.3 (41.0–43.5) | | 38.3 (37.1–39.6) | | 56.4 (55.2–57.7) | | 7.1 (6.4–7.7) | | 18.2 (17.2–19.2) | |  |
| Phalombe | 33.0 (31.8–34.2) | | 44.7 (43.5–46.0) | | 38.1 (36.9–39.3) | | 50.4 (49.1–51.6) | | 8.0 (7.3–8.7) | | 24.2 (23.1–25.2) | |  |
| Thyolo | 19.9 (18.9–20.9) | | 41.3 (40.0–42.5) | | 34.5 (33.3–35.7) | | 48.4 (47.1–49.7) | | 10.3 (9.6–11.1) | | 14.8 (13.9–15.7) | |  |
| Zomba City | 11.0 (10.2–11.8) | | 25.8 (24.7–26.9) | | 13.0 (12.1–13.8) | | 27.6 (26.5–28.8) | | 5.0 (4.4–5.5) | | 7.9 (7.2–8.6) | |  |
| Zomba Non-City | 14.8 (13.9–15.7) | | 31.0 (29.8–32.2) | | 27.9 (26.8–29.1) | | 43.1 (41.8–44.3) | | 9.4 (8.7–10.1) | | 12.4 (11.6–13.2) | |  |
